# In vitro and in vivo evaluation of cord blood hematopoietic stem and progenitor cells amplified with glycosaminoglycan mimetic

**DOI:** 10.1186/s13287-015-0267-y

**Published:** 2016-01-07

**Authors:** Lionel Faivre, Véronique Parietti, Fernando Siñeriz, Sandrine Chantepie, Marie Gilbert-Sirieix, Patricia Albanese, Jérôme Larghero, Valérie Vanneaux

**Affiliations:** Inserm, U 1160, Centre d’Investigation Clinique en Biothérapies, 75010 Paris, France; AP-HP, Hôpital Saint-Louis, Unité de Thérapie Cellulaire, Paris, F-75010 France; Département d’Expérimentation d’Animale, Université́ Paris Diderot, Institut Universitaire d’Hématologie, Hôpital Saint-Louis, Paris, F-75010 France; OTR3 Company, 4 rue Française, 75001 Paris, France; Université Paris Est Créteil, Université Paris Est, EA 4397 ERL CNRS 9215, Laboratoire CRRET, 61 Avenue du Général de Gaulle, 94010 Créteil, France; Université Paris Diderot, Sorbonne Paris Cité, U 1160, Paris, F-75010 France

**Keywords:** Hematopoietic stem cells, Expansion, Cord blood, Heparan sulfate, Glycosaminoglycan mimetic

## Abstract

**Background:**

Expansion protocols aim at both increasing the number of umbilical cord blood (UCB) hematopoietic stem cells and progenitor cells (HSPCs) and reducing the period of neutropenia in UCB HSPC graft. Because glycosaminoglycans (GAGs) are known to be important components of the hematopoietic niche and to modulate growth factor effects, we explored the use of GAG mimetic OTR4131 to potentiate HSPC’s in vitro expansion and in vivo engraftment.

**Methods:**

UCB CD34+ cells were expanded with serum-free medium, SCF, TPO, FLT3-lig and G-CSF during 12 days in the absence or the presence of increasing OTR4131 concentrations (0-100 μg/mL). Proliferation ratio, cell viability and phenotype, functional assays, migration capacity and NOD-scid/γc^-/-^ mice engraftment were assessed after expansion.

**Results:**

At Day 12, ratios of cell expansion were not significantly increased by OTR4131 treatment. Better total nucleated cell viability was observed with the use of 1 μg/mL GAG mimetic compared to control (89.6 % ± 3.7 % and 79.9 % ± 3.3 %, respectively). Phenotype analysis showed a decrease of monocyte lineage in the presence of OTR4131 and HSPC migration capacity was diminished when GAG mimetic was used at 10 μg/mL (10.9 % ± 4.1 % *vs.* 52.9 % ± 17.9 % for control). HSPC clonogenic capacities were similar whatever the culture conditions. Finally, in vivo experiments revealed that mice successfully engrafted in all conditions, even if some differences were observed during the first month. Three months after graft, bone marrow chimerism and blood subpopulations were similar in both groups.

**Conclusions:**

UCB HSPCs ex-vivo expansion in the presence of OTR4131 is a safe approach that did not modify cell function and engraftment capacities. In our experimental conditions, the use of a GAG mimetic did not, however, allow increasing cell expansion or optimizing their in vivo engraftment.

## Background

Umbilical cord blood (UCB) is an alternate source of both hematopoietic stem cells and progenitor cells (HSPCs) that is used to treat patients with hematologic diseases. Despite numerous advantages of UCB, such as easy access and lack of ethical issues, UCB HSPC transplantation has been hampered by the lower HSPC number compared with that in bone marrow or peripheral blood stem cells, leading to a longer period of post-transplant neutropenia and a delayed immunological reconstitution [1]. To overcome the limitation of cell quantity and maturity, many strategies have been proposed and developed to expand UCB HSPCs ex vivo and optimize the UCB transplantation process [2]. The advanced understanding of HSPC biology induced the development of numerous ex vivo amplification protocols based on growth factors and cocultivation. Using hematopoietic specific cytokines was the first and easiest method to amplify HSPCs [3, 4]. Based on this, we used a serum-free ex vivo culture protocol involving a cocktail of four selected cytokines (SCF, TPO, FLT3-lig, and granulocyte colony-stimulating factor (G-CSF)) developed by the Dazey laboratory and currently evaluated in clinical trials (NCT01034449), which have already demonstrated in vitro and in vivo relevant results [5]. An important milestone of such amplification protocols is not only to increase the amount of cells but also their survival quality, in order to optimize their in vivo engraftment in the medullary compartment.

The bone marrow stem cell niche is a complex structure, composed of cells, cytokines, and extracellular matrix (ECM) whose glycosaminoglycans (GAGs) are known to play an important role both in spatial organization and in interaction with cells and hematopoietic cytokines [6, 7]. In this site, the major GAGs are heparan sulfate (HS) and chondroitin sulfate (CS) [8]. Their use in ex vivo HSPC amplification protocols could participate in a better amplification process in terms of cell quality and quantity. Indeed, HS and CS are long polysaccharides composed of specific disaccharide building blocks, characterized by complex structures due to heterogeneity in glucosidic linkages, hydrophobic regions, and sulfatation patterns [9]. All properties of HS define the anionic interaction and hydrogen binding protein. Thus, HS could constitute a low-affinity receptor to heparin-binding proteins which could be necessary for signal transduction, such as described previously for syndecans on hematopoietic progenitor cells [10].

Owing to these properties, a family of chemically-engineered analogs of endogenous GAGs has been developed, named ReGeneraTing Agents^®^ (RGTA^®^; OTR3 SARL, Paris, France) [11, 12]. GAG mimetic syntheses are performed according to Good Manufacturing Practice grade, an essential point in human clinical issues. It was recently demonstrated that a specific GAG mimetic, namely OTR4131, is able to potentiate properties of different stem and progenitor cells: injection of this mimetic into mice was associated with mobilization of hematopoietic stem cells (HSCs) from the bone marrow to peripheral blood, according to an in vivo modification of the SDF-1 gradient [13].

According to the functional relevance of these GAG/cytokine interactions in HSPC homeostasis regulation, we investigated in the present study whether the association of a GAG mimetic could optimize the in vitro clinical UCB-derived HSPC amplification procedure. Moreover, whereas HSPC graft with intensive therapies (chemotherapy and/or total body irradiation) induced bone marrow lesions [14], we tested whether the presence of GAG mimetics during the UCB-derived HSPC expansion step could optimize the UCB HSPC in vivo engraftment.

## Methods

### HSPC purification, cryopreservation, and thawing

UCB units from normal full-term deliveries were obtained from Saint-Louis Hospital Cord Blood Bank (Paris, France) after mothers’ written informed consent, in accordance with health authorities (French Cord Blood Network, Paris, France). The study was approved by the Saint-Louis Hospital Biological Resource Center Scientific Council (authorized by the French Ministry of Research under number AC-2008-376). Mononuclear cells were separated with lymphocyte separation medium (Eurobio, Courtaboeuf, France). CD34^+^ HSPCs were isolated by supermagnetic microbead with a QuadroMAC Separator (Miltenyi Biotech, Paris, France) according to the manufacturer’s instructions. CD34 purified cells were kept frozen in liquid nitrogen and thawed on the day of amplification.

### RGTA**®**-OTR4131

RGTA®-OTR4131 mimetic was obtained from OTR3 SARL. RGTA^®^-OTR4131 is a dextran derivative, composed of about 250 glucosidic units, with carboxylate and sulfate degrees of substitution close to those of heparin, except for a lower degree of the acetyl group [11, 12]. When needed, OTR4131 was coupled with the fluorescent dye Alexa Fluor 647® (AF^647^-OTR4131) and image acquisition was made with Nikon BioStation IM (Nikon, Champigny sur Marne, France). OTR4131 compound was used during HSPC expansion at concentrations ranging from 0 to 100 μg/ml.

### Expansion protocol

Complete medium was composed of serum-free HP01 culture medium (Macopharma, Tourcoing, France) containing 100 ng/ml SCF, 20 ng/ml TPO, 100 ng/ml FLT3-Lig (all provided by Peprotech, Neuilly sur Seine, France), and 10 ng/ml Filgrastim (Zarzio^®^; Sandoz, Levallois-Perret, France), as described previously [15]. HSPC expansion was initiated by culturing 20 × 10^3^ CD45^+^/34^+^ viable purified cells per milliliter in complete medium at 5 % CO_2_ in water-saturated air for 6 days in 24-well plates. Cultured cells were then transferred into six-well plates (all from Becton Dickinson, Le Pont De Claix, France) for another 6 days, with fresh complete medium (4/1 v/v). Cell phenotype was performed at day 12 by flow cytometry analysis according to the antigen panel presented in Table 1. Cell expansion was expressed as the ratio between absolute cell numbers at day 12 divided by absolute cell numbers at day 0.

**Table 1:** Details of CD used for each cell population described

	Population	Panel	Percentage expressed in
In vitro analysis	Enriched HSC	CD45/34/90	CD45
	Myeloid progenitor	CD45/34^hi^/33	Cells
	Myeloid precursor	CD45/34^lo^/33	Cells
	Monocyte lineage	CD45/34^–^/33/14	Cells
	Neutrophil lineage	CD45/34^–^/14^–^/33	Cells
	B-lymphocyte progenitor	CD45/34/10/19	CD45
	B-lymphocyte lineage	CD45/10/19	CD45
	Megakaryocyte precursor	CD45/34/41/42	Cells
	Immature platelet	CD45/41/42	Cells
	Erythroid lineage	CD45/36/235a	Cells
	Immature erythrocyte	CD45^–^/36/235a	Cells
Mice analysis	Hematopoietic cells	CD45	Cells
	HSPCs	CD45/34	CD45
	Enriched HSC	CD45/34^hi^	CD45
	HSC	CD45/34/38^–^/90/45RA^–^	CD34
	MPP	CD45/34/38^–^/90^–^/45RA^–^	CD34
	CLP	CD45/34/10	CD34
	GMP	CD45/34/10^–^/38/135/45RA	CD34
	CMP	CD45/34/10^–^/38/135/45RA^–^	CD34
	MEP	CD45/34/10^–^/38/135^–^/45RA^–^	CD34
	T-lymphocyte progenitor	CD45/34/10^–^/19^–^/7	CD34
	B-lymphocyte progenitor	CD45/34/10/19/7^–^	CD34
	B-lymphocyte	CD45/34–/19/7^–^	CD45
	Myeloid progenitor	CD45/34^hi^/33	CD34
	Myeloid precursor	CD45/34^lo^/33/15	CD34
	Dendritic cell	CD45/34^–^/14/15^–^/11b/11c	CD45
	Monocyte	CD45/34^–^/33/14/15^–^	CD45
	Neutrophil	CD45/34^–^/33/14^–^/15	CD45
	Megakaryocyte precursor	CD45/34/41a/42	CD45
	Platelet	CD45^–^/41a/42	Cells
	Erythroid lineage	CD45/36/235a	CD45
	Erythrocyte	CD45^–^/235a	Cells

*CD* cluster of differentiation, *CLP* common lymphoid progenitor cells, *CMP* common myeloid progenitor cells, *GMP* granulocyte macrophage progenitor cells, *hi* high, *HSC* hematopoietic stem cells, *HSPC* hematopoietic stem cell and progenitor cell, *lo* low, *MEP* megakaryocyte erythroid progenitor cells, *MPP* multipotent progenitor cells

### Flow cytometry analysis

Cells were washed in phosphate-buffered saline and incubated for 30 minutes at 4 °C with a combination of monoclonal antibodies against human cell surface antigens as presented in Table 1. Antibodies were purchased as described: CD36 APC, CD14 PE-Cy7, CD42 PE, CD33 PE, CD34 APC, CD45 FITC (all from Becton Dickinson), CD34 Pacific Blue, CD45 Alexa Fluor 700, CD41 APC, CD90 APC, CD10 PE-Cy7, CD19 PE-Cy7 (all from Biolegend, Saint Quentin en Yvelines, France), and CD235a PE, 7-AAD (both from Beckmann Coulter, Villepinte, France). Cells were analyzed on a FACSCanto II flow cytometer (Becton Dickinson) using Diva software (version 6.1.3; Becton Dickinson).

### Migration assay

The migration assay was performed in 24-well culture plates with a 5 μm pore size Transwell (Corning, Tewksbury, Massachusetts, USA). SDF-1α (100 ng/ml; R&D Systems, Lille, France) was added to the lower chamber. Then 1 × 10^5^ cells per well, washed beforehand, were placed in the upper compartment and incubated at 37 °C and 5 % CO_2_. The percentage of cell migration was calculated after 6 hours.

### Colony-forming unit quantification

Clonogenicity of expanded cells was assessed by methylcellulose-based semisolid cultures according to the manufacturer’s protocol (MethoCult®; StemCell Technologies, Grenoble, France). Viable CD34^+^/CD45^+^ cells were seeded in duplicate in six-well culture plates at 300 cells/ml. Cells were incubated at 37 °C and 5 % CO_2_ and colony-forming units (CFUs) were scored using an inverted microscope after 14 days. Hematopoietic colonies were identified as follows: colonies containing both erythroid and myeloid cells (CFU-GEMM); colonies containing erythroid cells (CFU-E); myeloid colonies of pure granulocytic colonies (CFU-G); monocytic colonies (CFU-M); and colonies containing both granulocytes and monocytes (CFU-GM). Results were expressed as the CFU number per 1000 CD34^+^/CD45^+^ cells.

### Animal model

Immunodeficient NOD-scid/γc^–/–^ (NSG) mice (The Jackson Laboratory, Bar Harbor, Maine, USA) were bred and maintained in strict accordance with Directive 2010/63/UE. The protocol was approved by the Committee on the Ethics of Animal Experiments of the French Ministry of Agriculture (Permit Number: 02447.01). Eight-week old mice were irradiated with 2.25 Gy 24 hours before intravenous injection of 1 × 10^6^ cells expanded in the presence or absence of 1 μg/ml OTR4131, according to the protocol already described. The bone marrow, spleen, and thymus were harvested 1 and 3 months later, flushed, and crushed on a 100 μm strainer before flow cytometer analysis (*n* = 4 mice per time point) of different subsets of the hematopoietic population according to clusters of differentiation, as presented in Table 1. Chimerism was evaluated by the percentage of human CD45^+^ cells in different organs.

### Statistical analysis

Data are expressed as the mean ± standard deviation. Mean value comparisons were assessed by a *t* test on paired samples. All calculations were performed using MedCalc® (version 7.2.1.0; MedCalc Software, Ostend, Belgium). The reported *p* values are two-sided and significance was defined as *p* <0.05.

## Results

### In vitro evaluation of expanded HSPCs

**Fig. 1:**
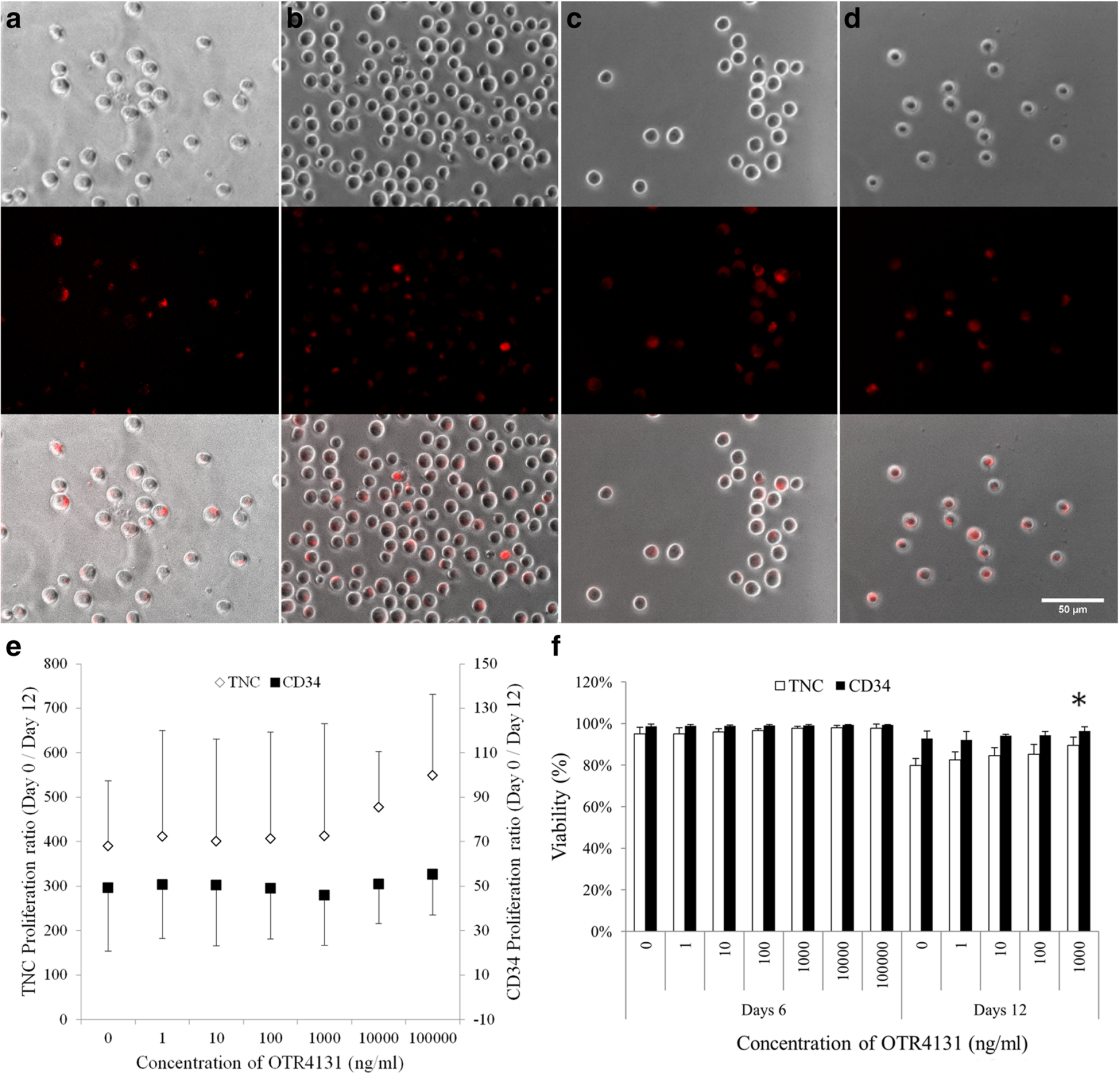
Effect of increasing concentrations of OTR4131 on HSPC proliferation and viability. **a**–**d** HSPC culture in the presence of 10 μg/ml AF^647^-OTR4131 at 3, 6, 9, and 12 days. *Upper panel*: phase contrast; *middle panel*: Cy5 staining; *lower panel*: merge (scale bar = 50 μm). **e** TNC and CD34^+^/CD45^+^ cell ratios between day 0 and day 12 (*n* = 9 for 0 to 1 μg/ml and *n* = 4 for 10 and 100 μg/ml OTR4131 concentrations). **f** TNC and CD34^+^/CD45^+^ cell viability at day 6 (*white bars*, *n* = 7) and day 12 (*dark bars*, *n* = 5). *p = 0.0024. *TNC* total nucleated cells

We first aimed at evaluating how GAG mimetic interacts with HSPCs. To this aim, 10 μg/ml AF^647^-OTR4131 was added to culture medium, and fluorescence was evaluated at days 3, 6, 9, and 12. AF^647^-OTR4131 fluorescent compound was shown to interact with HSPCs, and was found on the surface and in the cytoplasm of HSPCs whatever the time point considered. Intracellular staining was found to increase with time (Fig. 1a–d).

Purified UCB HSPC expansion was then performed, following a 12-day process. CD34^+^/CD45^+^ cell purity and viability before culture were 88.1 ± 7.7 % and 96.0 ± 1.9 %, respectively (*n* = 9). At day 12 the amplification ratios of total nucleated cells (TNC) and of CD34^+^/CD45^+^ cells were evaluated in the absence or the presence of increasing OTR4131 concentrations (from 1 ng/ml to 100 μg/ml). The mean TNC expansion ratio was 390.0 ± 147.1 for control, and ranged from 401.0 to 549.2 in the presence of OTR4131 (*p* >0.05 for all conditions). At the CD34^+^/CD45^+^ level, expansion in complete medium was 49.0 ± 28.3 at day 12, and between 45.7 and 55.1 at the tested OTR4131 concentrations (*p* >0.05, Fig. 1e).

After 6 days of expansion, TNC or CD34^+^/CD45^+^ cell viabilities were similar whatever the conditions. However, a higher TNC viability was obtained in the presence of 1 μg/ml OTR4131 after 12 days of expansion, with 89.6 ± 3.7 % compared with 79.9 ± 3.3 % in control conditions (*p* = 0.0024). CD34^+^/CD45^+^ viability followed the same trend in the presence of OTR4131, while not reaching significant statistical difference (Fig. 1f).

**Fig. 2:**
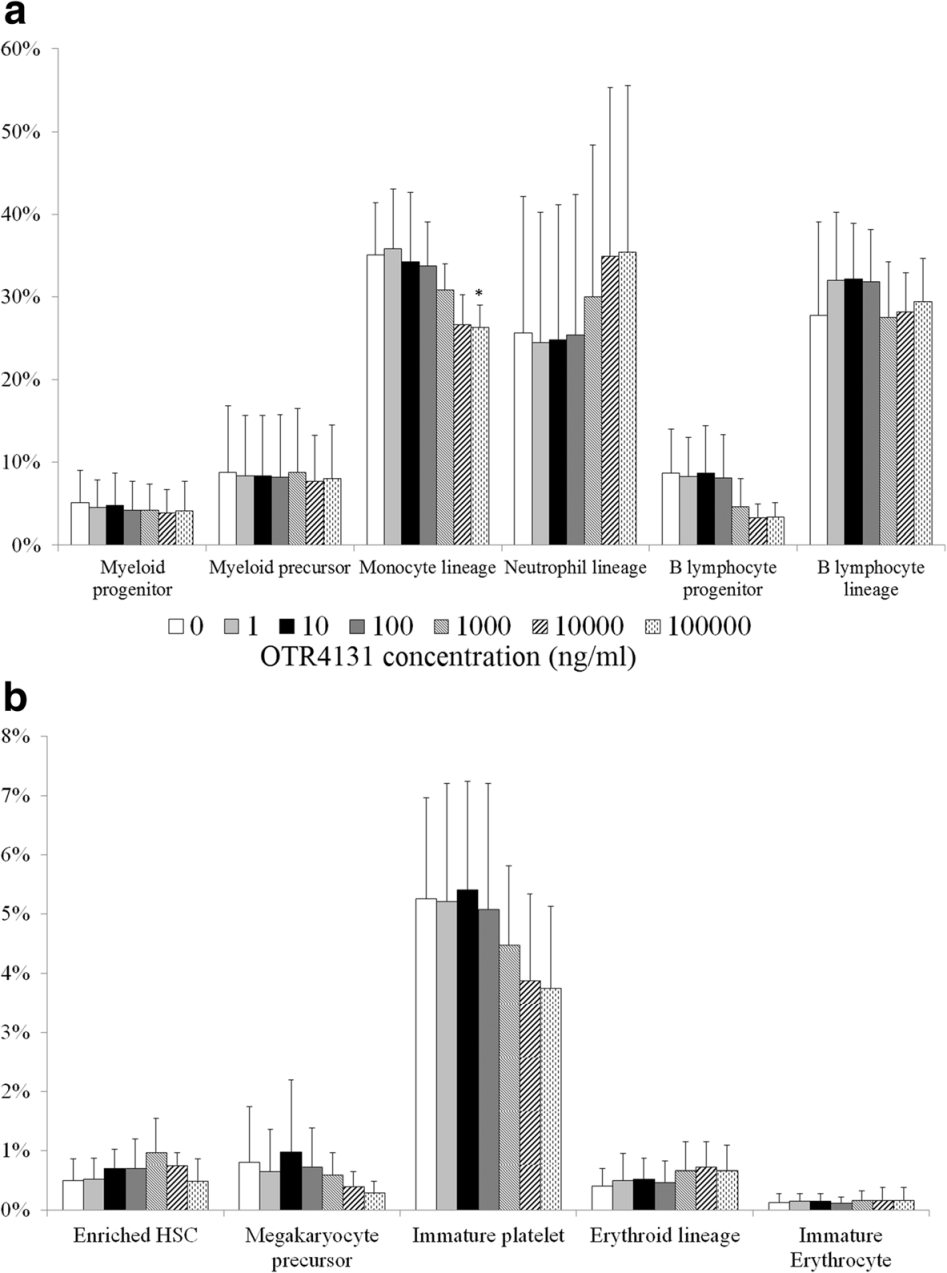
Flow cytometry analyses of expanded HSPCs at day 12 (*n* = 4). **a**. Percentages of myeloid and lymphoid lineage after expansion. **b**. Percentages of enriched HSC, megakaryocyte lineage and erythoid lineage after expansion. Each cell population was described following the cluster of differentiation presented in Table 1. *p <0.05. *HSC* hematopoietic stem cells

In order to determine the impact of OTR4131 on the outcome of cell phenotype during the amplification phase, cell phenotypes were analyzed after 12 days of expansion. Results are summarized in Fig. 2. The percentage of CD34^+^/CD45^+^ HSPCs was 14.1 ± 11.4 % in control conditions, and ranged from 5.6 to 31.3 % in the presence of OTR4131 (*p* >0.05 for all tested conditions). Within the CD34^+^/CD45^+^ cell population, the number of more immature CD34^+^/CD45^+^/CD90^+^ HSCs was also similar in the presence or absence of OTR4131 during the expansion protocol. Analysis of the committed myeloid subpopulations showed that only the monocyte percentage decreased in the presence of 100 μg/ml OTR4131 compared with control (35.1 ± 6.3 % vs. 26.3 ± 2.7 %, *p* = 0.04). The lymphoid lineage was not significantly modified whatever the experimental conditions, even if a slight decrease of B-lymphoid progenitors was observed with 10 μg/ml OTR4131. Platelet and erythroid lineages were also not affected.

**Fig. 3:**
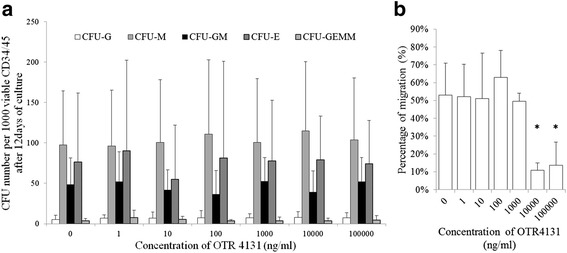
In vitro functional properties of expanded cells. **a** Number of CFU-GEMM, CFU-E, CFU-G, CFU-M, and CFU-GM per 1000 viable CD34^+^/CD45^+^ cells (*n* = 4, *p <0.05). Error bars correspond to the standard deviation. **b** Migration capacity, expressed as the percentage of migration of total cells in front of a SDF-1 gradient (*p* = 0.0038 and *p* = 0.012 respectively, *n* = 4). *CFU-E* colony-forming units of erythrocytes, *CFU-G* colony-forming units of granulocytes, *CFU-GEMM* colony-forming units of granulocytes, erythrocytes, monocytes, and megakaryocytes, *CFU-GM* colony-forming units of granulocytes and monocytes, *CFU-M* colony-forming units of monocytes

HSPC clonogenic capacities were also assessed at the end of the expansion protocol. After 14 days of semisolid culture of 1000 viable CD34^+^/CD45^+^ cells, the numbers of CFU-G, CFU-M, CFU-GM, CFU-E, and CFU-GEMM were not significantly different between control and increasing OTR4131 concentration (Fig. 3a), thus suggesting that HSC expansion in the presence of GAG mimetic does not affect HSPC differentiation properties.

Interestingly, high OTR4131 concentrations were shown to diminish cell migration capacities toward the SDF-1 gradient. At 10 and 100 μg/ml OTR4131, the percentages of migration were 10.9 ± 4.1 % and 13.7 ± 12.9 %, respectively, compared with 52.9 ± 17.9 % for controls (*p* = 0.0038 and *p* = 0.0120, respectively, Fig. 3b). This effect was not observed when GAG mimetic was used at a concentration of 1 μg/ml, for which HSPC migration was similar to that of controls.

### In vivo engraftment of expanded HSPCs

Engraftment efficiency of expanded HSPCs and chimerism in mice hematopoietic organs were compared between controls and cells expanded with 1 μg/ml OTR4131. This concentration was chosen because 1 μg/ml OTR4131did not affect the main tested parameters (amplification ratio, phenotype, migration, and clonogenicity) and tended to increase cell viability.

**Fig. 4:**
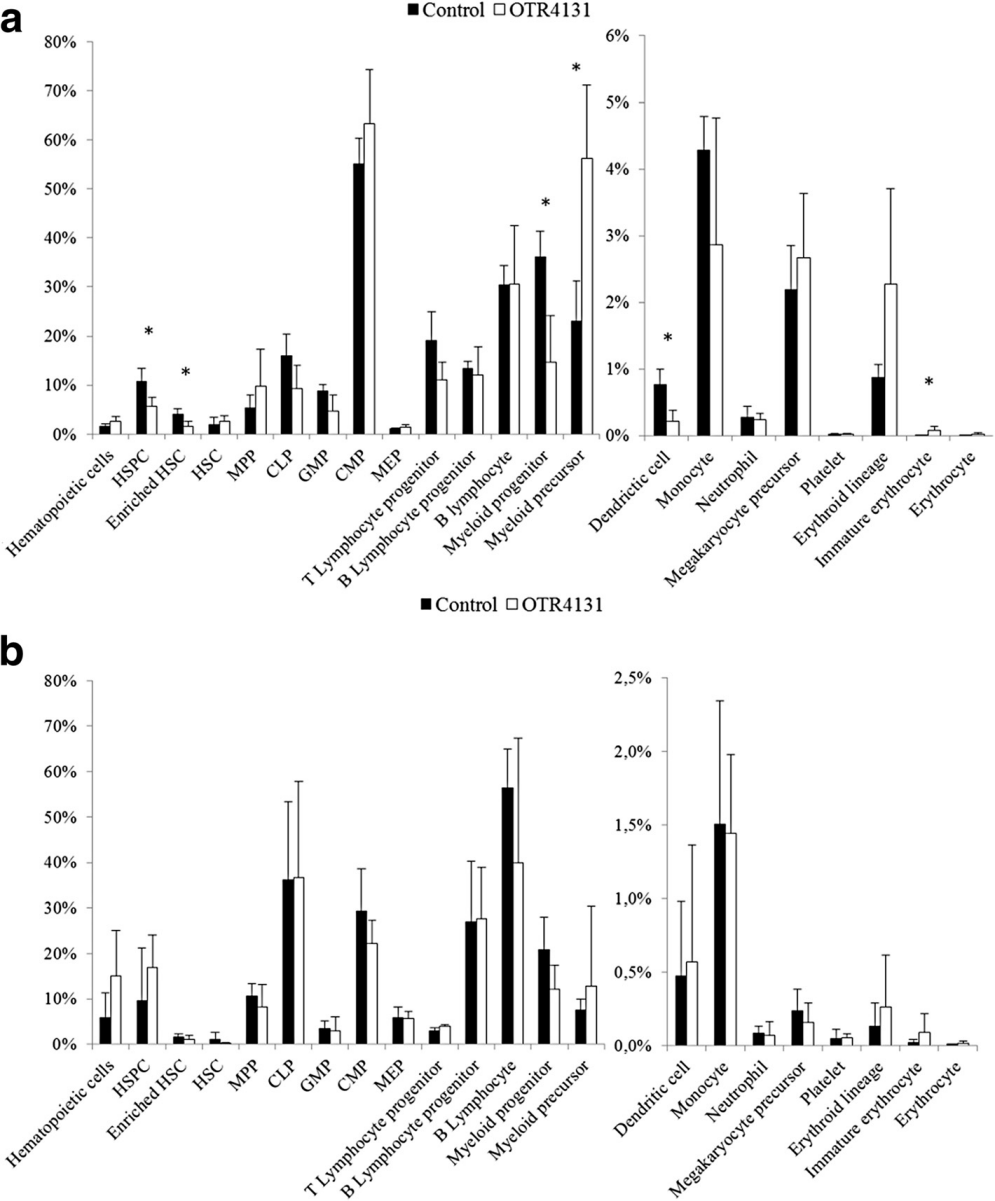
Bone marrow NSG mice reconstitution after transplantation of expanded HSPCs. **a** One month after transplantation, human hematopoietic cells were characterized in mice bone marrow by flow cytometry. Cell markers are presented in Table 1 (*n* = 4). *p <0.05. **b** The same analyses were performed 3 months after transplantation (*n* = 3). *CLP* common lymphoid progenitor cells, *CMP* common myeloid progenitor cells, *GMP* granulocyte macrophage progenitor cells, *hi* high, *HSC* hematopoietic stem cells, *HSPC* hematopoietic stem cell and progenitor cell, *lo* low, *MEP* megakaryocyte erythroid progenitor cells, *MPP* multipotent progenitor cells

Data of bone marrow reconstitution 1 month after transplantation are summarized in Fig. 4. The percentages of chimerism (CD45^+^ cells) were no different between controls (1.6 ± 0.5 %) and 1 μg/ml OTR4131 (2.3 ± 1.0 %) expanded HSPCs. However, HSPCs and HSCs (defined as CD45^+^/CD34^high^ cells) were lower in the OTR4131 group than in controls, with 5.6 ± 1.9 % vs. 10.8 ± 2.6 % (*p* = 0.0174) and 1.7 ± 1.0 % vs. 4.1 ± 1.1 % (*p* = 0.0181), respectively. No significant statistical differences were observed between the two groups regarding the various immature progenitors, such as multipotent progenitor cells, common myeloid progenitor cells, common lymphoid progenitor cells, megakaryocyte erythroid progenitor cells, and granulocyte macrophage progenitor cells. Similar results were obtained for lymphoid and megakaryocyte lineages. Some differences were obtained for myeloid lineage, however, with a decreased percentage of myeloid progenitors (CD45^+^/CD34^high^/CD33^+^) in the OTR4131 group (14.7 ± 9.4 %) compared with controls (36.2 ± 5.2 %) (*p* = 0.0072), associated with an increased percentage of myeloid precursor (CD45^+^/CD34^low^/CD33^+^/CD15^+^): 56.3 ± 15.5 % and 23.1 ± 8.1 %, respectively (*p* = 0.0080). Mature myeloid cell, neutrophil, and dendritic cell percentages were not modified, while monocytes were decreased in the OTR4131 group (0.2 ± 0.2 %) compared with the control group (0.8 ± 0.2 %) (*p* = 0.0077). Lastly, the erythroid lineage increased in the OTR4131 group, particularly for immature erythroid cells (CD45^–^/CD36^+^/CD235^+^), with percentages of 0.086 ± 0.061 % compared with 0.010 ± 0.003 % in the control group (*p* = 0.0477).

The bone marrow, thymus, and spleen chimerisms (CD45^+^ cells) were analyzed in mice 3 months after expanded cell transplantation. No statistical difference was shown between controls and OTR4131 expanded cells, with a percentage of human cells of 5.8 ± 5.4 % vs. 15.0 ± 10.1 % in the bone marrow, of 21.2 ± 36.0 % vs. 11.7 ± 6.6 % in the thymus, and of 1.6 ± 0.8 % vs. 3.4 ± 2.0 % in the spleen, respectively. These results were consistent with similar percentages of hematopoietic lineages in the bone marrow in both groups (Fig. 4). Thymus and spleen explorations also showed that mice equally reconstitute progenitor and/or mature lineages (data not shown).

## Discussion

Since the first definition of the stem cell niche introduced by Schofield [16], the knowledge of the microenvironment role toward hematopoiesis has evolved extensively. Cytokines and proteoglycans have been shown to play an important role in HSPC maintenance and differentiation. The role of proteoglycans has been extended to support endogenously expressed hematopoietic cytokines on the surface of stromal cells, in ECM, and in solution [17, 18]. Moreover, a high fraction of total GAGs synthesized by stromal or hematopoietic cells is present in solution rather than on the stromal layer [19–21]. In the bone marrow niche, it is well known that GAGs play an important role in spatial organization and in interaction with hematopoietic cytokines [6, 7, 22]. For example, it was demonstrated that Glypican-3 proteoglycan is involved in HSC homing and maintenance mechanism, implicating interactions between the chemokine CXCL12 and its receptor CXCR4 [23]. Moreover, the relevance of GAG in the homeostasis of medullar microenvironment is attested by the demonstration that heparanase activity regulates retention and proliferation of primitive Sca-1/c-Kit/Lin^–^ cells in mice bone marrow, whereas a reduction in the overall leukocyte cellularity bone marrow is observed. This suggests an important role of heparanase, and accordingly of HS, in hematopoietic progenitor cell regulation and control of the primitive cell pool size [24]. More recently, Saez et al*.* [25] demonstrated that inhibition of HS production in bone marrow osteolineage Mx1 stromal cells results in a disruption of HSC interactions within the niche and their egress into the peripheral circulation.

HSs, more particularly, are part of GAG subsets that can maintain HSPCs for in vitro long-term culture [18]. Several studies have also demonstrated an effect of heparin or noncharacterized human stromal-derived HS on different kinds of hematopoietic progenitors. Their presence during ex vivo amplification protocols has thus improved both megakaryocytic maturation and the amplification ratio [26–28]. For all these reasons, we hypothesized that OTR4131, a sulfated GAG mimetic, could have an interest in UCB HSPC amplification protocols.

These protocols were initially developed to overcome some limitations of UCB graft, such as delayed neutropenia and the need for transfusion caused by low number and immaturity of HSPCs [1, 2]. According to the World Health Organization, more than 25 clinical trials have currently been initiated.

The present study deals with the GAG mimetic OTR4131, a well-characterized synthetic polymer RGTA®. OTR4131 was here used for ex vivo amplification of UCB-derived HSPCs during 12 days. First of all, we observed an important interaction between fluorescent OTR4131 (at 10 μg/ml) and HSPCs from 2 days of culture. Below this concentration, the fluorescent OTR4131 was not detectable with our fluorescent microscope. Concerning the amplification ratio, no difference was observed in the presence or absence of OTR4131, except for a better TNC viability at 1 μg/ml OTR4131. A similar increase of viability was observed on rat skin fibroblasts with 1 μg/ml OTR4120, another GAG mimetic compound. In this study, Yue et al. [29] concluded that this effect was related to a regulation of the premitochondrial death cascade with an inhibition of lysosomal cathepsin activities and a protection of the lysosome from membrane disruption. In that work, the RGTA^®^ effect was associated with a reduction of intracellular reactive oxygen species levels and an inhibition of mitochondrial membrane potential collapse [29]. Moreover, we observed a decrease in the migration of amplified HSPCs with OTR4131, which could be explained by the elevated quantity of OTR4131 on the HSPC surface, blocking SDF-1α chemoattraction by a sequestration effect [30]. Based on their phenotype or their clonogenic capacity, the composition of amplified HSPC populations was similar with or without OTR4131, except for an isolated decrease in monocyte lineage.

Furthermore, according to in vitro results of 1 μg/ml OTR4131 mimetic, the effect on NSG graft experiments was limited after 1 month: the different populations of HSPCs (CD34^+^ or CD34^+high^) were statistically diminished in the OTR4131 group; and we observed an increase in the percentage of myeloid precursor cells and immature fraction of erythrocyte lineage. This modification was not stabilized in long-term graft whatever the lineage, and we therefore cannot conclude about a lasting change. These data suggest that GAGs probably impact cell differentiation at the level of long-term culture initiating cells (LTC-ICs) rather than at that of precursor or immature HSC. This hypothesis may also explain the modification observed on myeloid precursors, progenitors, and mature cells. These results are consistent with those already reported by Gupta et al. [17] during LTC-IC in vitro culture in the presence of various GAGs.

Even if the benefits of this specific mimetic on amplification protocols were too slight, however, we will discuss the interest of screening new compounds with more effective structural and functional features that would present an interest for in vitro or in vivo further uses. Accordingly, in the literature, GAG effects are linked to their structural signature. Gupta et al. [17, 18] compared the sulfatation profile of HS derived from a hematopoiesis supportive and nonsupportive cell line and found that a fine pattern of sulfate substitution is necessary to bind to hematopoietic cytokines such as interleukin-3, macrophage inflammatory protein-1α, and thrombospondin. Moreover, sulfatation pattern alteration has been found to reduce the ability of heparin, a more extensively sulfated form of HS, to maintain LTC-ICs [18, 31]. These structural characteristics provide specific binding properties to proteins. Thereby GAG-bind proteins are protected against proteases and interactions with their receptors are facilitated [9, 32, 33]. For example, fine characterization of the binding of heparin to G-CSF demonstrated the importance of sulfate groups since 2,3-*O*-sulfate groups are more important than *N*-sulfate groups in heparin–G-CSF interaction [34]. Heparin-induced leukocytosis requires glucosamine 6-*O*-sulfation and is caused by blockade of L-selectin-mediated, P-selectin-mediated, and CXCL12-mediated leukocyte trafficking [35]. Finally, it was demonstrated that accumulated overly sulfated extracellular HS, in patients suffering from Mucopolysaccharidosis I, alters cytokine gradient formation since they abnormally bind and sequester CXCL12. This induces limited hematopoietic migration and engraftment, providing a potential mechanism for the limited scope of HSPC transplantation in Hurler syndrome [36].

## Conclusion

Taking into consideration the development of ex vivo amplification UCB-derived HSPC strategy, the aim of this study was to potentiate a protocol based on a serum-free medium added with heparin-binding cytokines with a GAG mimetic named OTR4131. In our experimental conditions, GAG mimetic in medium during cell amplification seems not to be the best strategy. However, it could be interesting to design and screen several GAG mimetics with different sulfatation patterns that could be optimized to improve HSPC amplification.

## Abbreviations

AF^647^: Alexa Fluor 647
CFU: Colony-forming unit
CFU-E: CFU of erythrocyte
CFU-G: CFU of granulocyte
CFU-GEMM: CFU of granulocyte, erythrocyte, monocyte, and megakaryocyte
CFU-GM: CFU of granulocyte and monocyte
CFU-M: CFU of monocyte
CS: Chondroitin sulfate
ECM: Extracellular matrix
GAG: Glycosaminoglycan
G-CSF: Granulocyte colony-stimulating factor
HS: Heparan sulfate
HSC: Hematopoietic stem cell
HSPC: Hematopoietic stem cell and progenitor cell
LTC-IC: Long-term culture initiating cell
NSG: NOD-scid/γc^–/–^
RGTA®: ReGeneraTing Agents®
TNC: Total nucleated cells
UCB: Umbilical cord blood
